# Correlation between PPARγ protein expression level in granulosa cells and pregnancy rate in IVF program

**Published:** 2012-03

**Authors:** Mehdi Sahmani, Reza Najafipour, Laya Farzadi, Ebrahim Sakhinia, Masoud Darabi, Vahideh Shahnazi, Amir Mehdizadeh, Maghsod Shaaker, Mohammad Noori

**Affiliations:** 1 Department of Biochemistry and Clinical Laboratories, Faculty of Medicine, Tabriz University of Medical Sciences, Tabriz, Iran.; 2 *Department of Clinical Biochemistry and Molecular and Cellular Research Center, Ghazvin University of Medical Sciences, Qazvin, Iran.*; 3 Women’s Reproductive Health Research Center, Alzahra Hospital, Tabriz University of Medical Sciences, Tabriz, Iran.; 4 Drug Applied Research Center, Tabriz University of Medical Sciences, Tabriz, Iran.

**Keywords:** *In-vitro fertilization*, *Peroxisome proliferative-activated receptor γ*, *Granulosa cells*, *Pregnancy rate*

## Abstract

**Background:** Peroxisome proliferative-activated receptors (PPARs) are nuclear receptors that involved in cellular lipid metabolism and differentiation. The subtype γ of the PPAR family (PPARγ) plays important roles in physiologic functions of ovaries.

**Objective:** To determine correlation between PPARγ protein level in granulosa cells and pregnancy rate in women undergoing in-vitro fertilization (IVF) treatment.

**Materials and Methods:** In this cross-sectional study**, **twenty-five samples of granulosa cells were collected from women referred to an IVF treatment center. PPARγ protein expression level in granulosa cells was determined in comparison with β-actin level as control gene with Western blot test. Laboratory pregnancy was determined by a rise in blood β-hCG level fourteen days after embryo transfer. Correlation analyses were used to test for associations between the oocytes and pregnancy occurrence as outcome variables and PPARγ protein expression level.

**Results: **Correlation analysis indicated that there was no significant relationship between granulosa cells PPARγ protein level with IVF parameters including number of matured oocytes and the ratio of fertilized to matured oocytes. Comparison of granulosa cells PPARγ protein level with positive and negative laboratory pregnancy revealed also no significant relationship.

**Conclusion: **According to the results of this study, PPARγ protein level in granulosa cells could not be directly correlated to the success rate of IVF.

## Introduction

Different studies show that various factors may potentially affect IVF success rate (1). PPARγ (peroxisome proliferative- activated receptor gamma) has been known as an important regulatory factor in fertility (2). PPARγ gene is located on the short arm of the chromosome 3 and consists of 9 exons with more than 100 kb (2). PPARγ is a member of intracellular (nuclear) receptors and belongs to the steroidal /thyroidal receptor family (3). 

After ligand to receptor binding and forming active complex, receptor can attach to some portions of DNA, called PPAR response element (PPARE) to modify gene expression level (3). Some natural ligands such as unsaturated fatty acids or synthetic agents act as activator of PPAR family (3, 4). Subgroup γ of PPARs plays important roles in gluconeogenesis, biosynthesis, deposition and catabolism of lipids. PPARγ increase insulin sensitivity and as a result decrease blood glucose in patients with type II diabetes mellitus (3). It has been shown that decreased PPARγ activity relates to decreasing hormone biosynthesis in ovaries (5). Antoine et al (6) showed that PPARγ acts as modifier gene in general population in contrast to patients with polycystic ovary syndrome (PCOS). Expression of this gene has been detected in different stages of folliculogenesis and the highest expression level has been reported after follicle releasing and LH surge (4). 

Recent studies showed that PPARγ plays an important role in ovary tissue changes during ovulation, as well as facilitation of ovulation in the every cycle of menstruation in mammals (2). Fan *et al* (7) showed that PPARγ blocks androgen conversion to estradiol by inhibiting the expression of aromatase. The production of estradiol by ovaries plays an important role in uterine preparation for zygote implantation. Thus, any disturbances in this hormone may cause decrease of implantation rate and infertility (6). Study of Shah *et al* showed that PPARγ agonists decrease vascular endothelial growth factor (VEGF) production by human luteinized granulosa cells in-vitro without any adverse impact on the development of cultured murine embryos (8). 

PPARγ is thought to be involved in negative regulation of trophoblast invasion into the decidua (9). Thus, the effects of PPARγ on progesterone production may be dependent on the cell type, differentiation and cycle stage (10). Cui *et al* (10) observed that animal model in which PPARγ gene is deleted had normal ovulation in spite of decreased secretion rate of progesterone and as a result profound decreased rate of zygote implantation rate. As the relationship between PPARγ and infertility is controversial, we decided to organize this study aiming to examine correlation between PPARγ protein level in granulosa cells and pregnancy rate in women undergoing in-vitro fertilization (IVF) treatment.

## Materials and methods

We designed a cross-sectional study and gained the agreement of ethical committee of Tabriz University of Medical Science. Twenty-five women (32.0±5.6 years of age, BMI 23.8±1.6 kg/m^2^) who were candidate for IVF, with unexplained infertility after a standard infertility evaluation, entered the study. 

All of them had healthy non-smoker husbands with normal spermogram. History of uterine disorders, poor ovarian response, endocrine and inflammatory diseases like thyroidal, adrenal, immune system and sex hormones disturbances were criteria’s for exclusion from the study. Ovarian stimulation was achieved with a long GnRH agonist (decapeptyl; Debio Pharm, Geneva, Switzerland)/ FSH-long down regulation protocol. Controlled ovarian stimulation was started with recombinant human follicle stimulating hormone (rFSH, Gonal-F; Serono, Switzerland) at the third day of menstrual cycle. Intramuscular hCG (1000IU, Choriomon, Meizler, Brazil) was administered when sonography revealed that average diameter of 3 preovulatory follicle had approached 18-20mm.

Oocyte retrieval was done 36 hours after hCG administration by vaginal ultrasound-guided puncture of the ovarian follicles. The collected oocytes were incubated in 37ºC with 5% CO2 for 4 hours and then were used for IVF. A maximum of three embryos were transferred at 4-8 cells stages after 48 hours, using ultrasound guidance. Chemical pregnancy assessed by β-hCG test, 14 days after embryo transfer.

Granulosa cells were isolated from follicular fluid by centrifugation and were placed in lysis buffer containing anti-protease cocktail. Protein concentration in the supernatant of lysed cells was measured using Bradford's colorimetric method with reference to BSA standards (Bio-Rad). Western blot analysis was performed according to the standard procedures (Bio-Rad, Richmond, CA). 

Briefly, 30 µg of whole cell extract were separated by SDS-PAGE. After electro-transfer to Immobilon-P membrane (Millipore, Bedford, MA), the blots were blocked with 3% skim milk and subjected to Western blot analysis with either polycolonal anti-PPARγ or anti-β-actin (Abcam, Cambridge, MA). Immuno reactive bands were detected by enhanced ECL (Amersham Bioscience). For quantification, the developed films were scanned and pixel intensity of PPARγ signal was normalized against β-actin for each individual pixel.

**Statistical analysis**

Correlation analysis was performed by Pearson correlation coefficient to test for associations between the oocytes and pregnancy occurrence as outcome variables and PPARγ protein expression level. A p-value of less than 0.05 was considered statistically significant. All analyses were made using the Statistical Package for the Social Sciences (SPSS Inc. Chicago, IL) software, version 11.0. 

## Results

**Figure 1:**
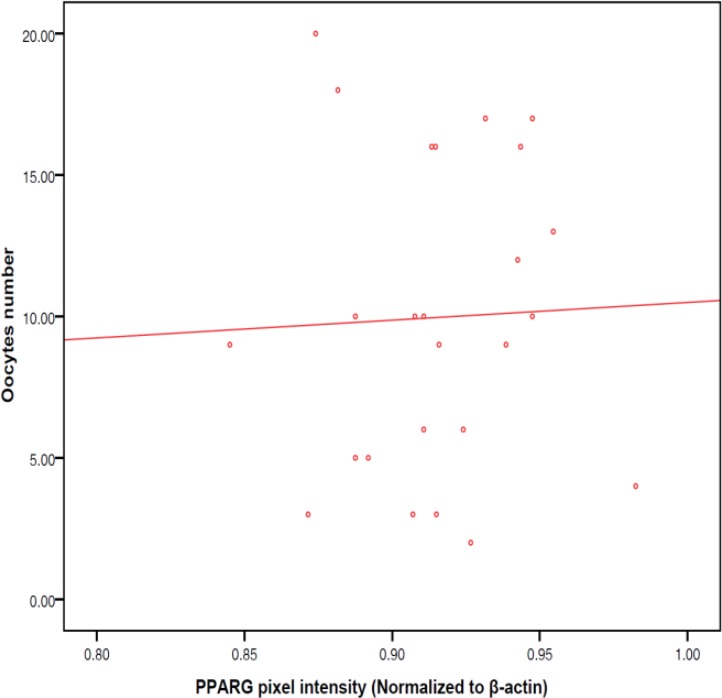
Relationship between PPARγ protein level in granulosa cells and number of mature oocytes (r=0.04, p=0.87).

**Figure 2:**
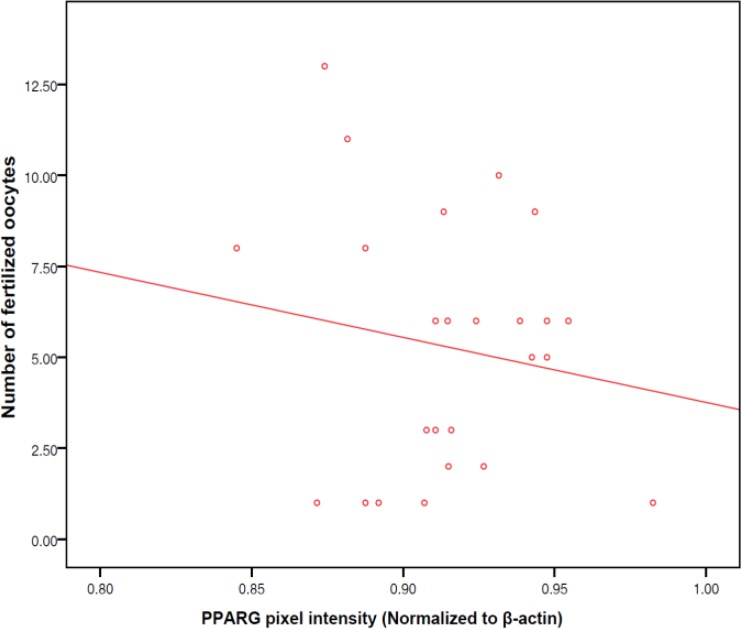
Relationship between PPARγ protein level in granulosa cells and number of fertilized oocytes (r=-0.10, p=0.45).

**Figure 3:**
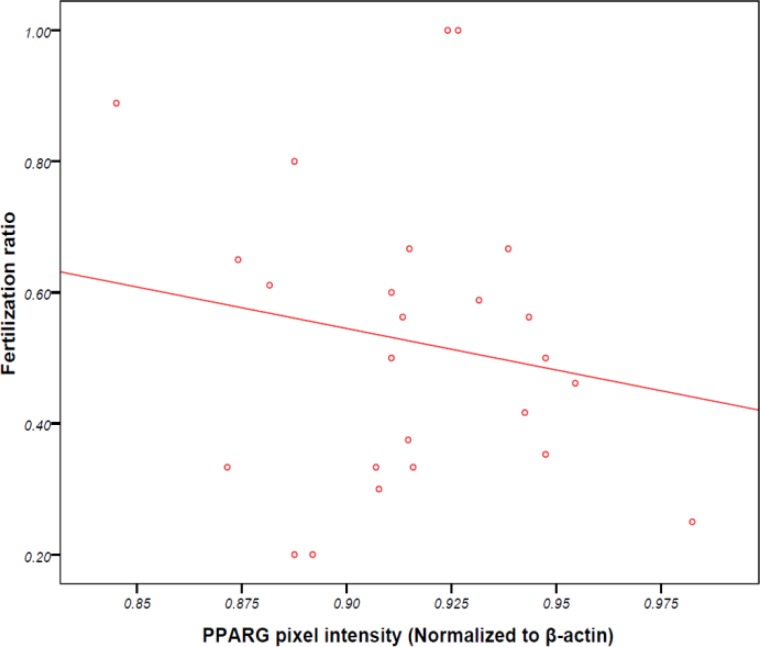
Relationship between PPARγ protein level in granulosa cells and ratio of fertilized to matured oocytes (r=-0.11, p=0.42).

**Figure 4:**
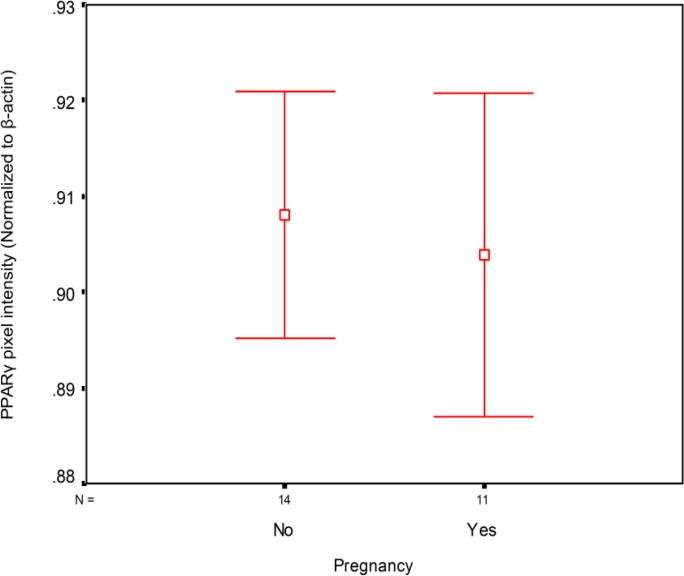
Comparison of PPARγ protein level in granulosa cells on the basis of laboratory pregnancy occurrence (p=0.68).

Average numbers of matured and fertilized oocytes were 10.6±5.2 and 6.1±3.4, respectively. The rate of biochemical pregnancy was 35.9%. No significant relationship were observed between PPARγ protein expression level in granulosa cells and number of mature oocytes (r=0.04, p=0.87), number of fertilized oocytes (-0.10, p=0.45) or the ratio of fertilized to mature oocytes (r=-0.11, p=0.42; Figures 1-3). As shown in figure 4, no significant relationship were found between PPARγ protein level in granulosa cells and laboratory pregnancy rate, based on serum βhCG levels.

## Discussion

Study of IVF treatment success can help us to better understand pathogenesis, diagnosis and management of infertility in women. Studies of Forment *et al* (4) showed that TZDs (Thiazolidine diones) as PPARγ ligands have no effect on the pituitary secretion of FSH, LH and prolactine in-vitro (11). They also showed that one of the known functions of PPARγ is modification of cell proliferation and differentiation. 

It also stimulates follicular differentiation rate. The semi-quantified data of our study from Western blot showed no relation between the PPARγ level and IVF success rate. Oocytes quality is an important factor in fertility progress. Different in vitro studies have shown that different genetic problems especially in PPARγ gene play important roles in fertility success (10). Maturation of oocytes depends on their interaction with the surrounding granulosa cells. There are also some evidence indicating PPARγ affects on granulosa cells and folliculogenesis function (12).

Some studies have reported controversial findings. Lovekamp-Swan *et al* (13) showed that mice with PPARγ gene knock-down had normal follicles and these follicles could cause yellow body development. This study supposed that PPARγ is not necessary for follicle activity. Nevertheless, in these animals the rate of implantation decreased, suggesting that PPARγ may have role, at least in part, in regulation of oocyte maturation through exerting negative regulation on growth and differentiation of follicles. 

Results of Lovekamp-Swan *et al* (13) study supported this hypothesis that PPARγ is a negative regulator in cumulus cell growth in rats and inhibits their unlimited growth. Although this study has shown a possible effect of PPARγ on growth of follicular and leuteal phase, its level had not been controlled at different stages. In comparison with rat, mRNA level and PPARγ gene expression level in sheep at the time of follicle growing was severely decreased which its cause still is not clear (14).

In the current study no relationship was detected between PPARγ protein expression level in granulosa cells, IVF parameters and pregnancy rate. However, it is possible that the function and importance of PPARγ is different in various clinical stages of pregnancy. Moreover, changes in mRNA modification and post-translational modification on the PPARγ protein are the effective mechanisms of PPARγ cellular activity and could affect independently on the PPARγ biological activity. Because of the multifactorial nature of IVF success, it seems that assessing the level of PPARγ level in granulosa cells at different stages of fertility would be very important to determine the role of PPARγ in fertility.

The small number of patients in this report may seem as a limitation. However, only infertile couples with no interfering health conditions were entered the study. Western blotting may not be technically sensitive enough to detect small alterations in the PPARγ protein expression levels. On the other hand, posttranslational modification contributes to the modulating activity of PPARγ without affecting the overall level of PPARγ protein (15). So, it would be desirable to measure the actual activity of PPARγ to more clarify its relation to IVF outcome.

We could not find any relationship between PPARγ protein level in granulosa cells and parameters of IVF and pregnancy. It was concluded that PPARγ protein level in granulosa cells possibly could not be directly correlated to the success of IVF. 

## References

[1] Younglai EV, Holloway AC, Foster WG (2005). Environmental and occupational factors affecting fertility and IVF success. Hum Reprod Update.

[2] Minge CE, Robker RL, Norman RJ (2008). PPAR Gamma: Coordinating Metabolic and Immune Contributions to Female Fertility. PPAR Res.

[3] Chinetti-Gbaguidi G, Fruchart JC, Staels B (2005). Role of the PPAR family of nuclear receptors in the regulation of metabolic and cardiovascular homeostasis: new approaches to therapy. Curr Opin Pharmacol.

[4] Froment P, Gizard F, Defever D, Staels B, Dupont J, Monget P (2006). Peroxisome proliferator-activated receptors in reproductive tissues: from gametogenesis to parturition. J Endocrinol.

[5] Komar CM, Braissant O, Wahli W, Curry TE Jr (2001). Expression and localization of PPARs in the rat ovary during follicular development and the periovulatory period. Endocrinology.

[6] Antoine HJ, Pall M, Trader BC, Chen YD, Azziz R, Goodarzi MO (2007). Genetic variants in peroxisome proliferator-activated receptor gamma influence insulin resistance and testosterone levels in normal women, but not those with polycystic ovary syndrome. Fertil Steril.

[7] Fan W, Yanase T, Morinaga H, Mu YM, Nomura M, Okabe T (2005). Activation of peroxisome proliferator-activated receptor-gamma and retinoid X receptor inhibits aromatase transcription via nuclear factor-kappaB. Endocrinology.

[8] Shah DK, Menon KM, Cabrera LM, Vahratian A, Kavoussi SK, Lebovic DI (2010). Thiazolidinediones decrease vascular endothelial growth factor (VEGF) production by human luteinized granulosa cells in vitro. Fertil Steril.

[9] Barak Y, Sadovsky Y, Shalom-Barak T (2008). PPAR Signaling in Placental Development and Function. PPAR Res.

[10] Cui Y, Miyoshi K, Claudio E, Siebenlist UK, Gonzalez FJ, Flaws J (2002). Loss of the peroxisome proliferation-activated receptor gamma (PPARgamma) does not affect mammary development and propensity for tumor formation but leads to reduced fertility. J Biol Chem.

[11] Rees WD, McNeil CJ, Maloney CA (2008). The Roles of PPARs in the Fetal Origins of Metabolic Health and Disease. PPAR Res.

[12] Faut M, Elia EM, Parborell F, Cugnata NM, Tesone M, Motta AB (2011). Peroxisome proliferator-activated receptor gamma and early folliculogenesis during an acute hyperandrogenism condition. Fertil Steril.

[13] Lovekamp-Swan T, Chaffin CL (2005). The peroxisome proliferator-activated receptor gamma ligand troglitazone induces apoptosis and p53 in rat granulosa cells. Mol Cell Endocrinol.

[14] Froment P, Fabre S, Dupont J, Pisselet C, Chesneau D, Staels B (2003). Expression and functional role of peroxisome proliferator-activated receptor-gamma in ovarian folliculogenesis in the sheep. Biol Reprod.

[15] van Beekum O, Fleskens V, Kalkhoven E (2009). Posttranslational modifications of PPAR-gamma: fine-tuning the metabolic master regulator. Obesity (Silver Spring).

